# Biomaterial-supported MSC transplantation enhances cell–cell communication for spinal cord injury

**DOI:** 10.1186/s13287-020-02090-y

**Published:** 2021-01-07

**Authors:** Bin Lv, Xing Zhang, Jishan Yuan, Yongxin Chen, Hua Ding, Xinbing Cao, Anquan Huang

**Affiliations:** 1 Department of Orthopedics, The Affiliated People’s Hospital of Jiangsu University, Zhenjiang, 212002 Jiangsu Province China; 2 Department of Trauma and Reconstructive Surgery, RWTH Aachen University Hospital, 52074 Aachen, Germany; 3 Department of Orthopedics, The Affiliated Hospital of Jiangsu University, Zhenjiang, 212000 Jiangsu Province China; 4 Department of Orthopedics, The Affiliated Suzhou Hospital of Nanjing Medical University, Suzhou Municipal Hospital, Suzhou, 215000 Jiangsu Province China

**Keywords:** Biomaterial, MSC transplantation, Spinal cord injury, Combinatorial therapy, Neuroinflammation

## Abstract

The spinal cord is part of the central nervous system (CNS) and serves to connect the brain to the peripheral nervous system and peripheral tissues. The cell types that primarily comprise the spinal cord are neurons and several categories of glia, including astrocytes, oligodendrocytes, and microglia. Ependymal cells and small populations of endogenous stem cells, such as oligodendrocyte progenitor cells, also reside in the spinal cord. Neurons are interconnected in circuits; those that process cutaneous sensory input are mainly located in the dorsal spinal cord, while those involved in proprioception and motor control are predominately located in the ventral spinal cord. Due to the importance of the spinal cord, neurodegenerative disorders and traumatic injuries affecting the spinal cord will lead to motor deficits and loss of sensory inputs.

Spinal cord injury (SCI), resulting in paraplegia and tetraplegia as a result of deleterious interconnected mechanisms encompassed by the primary and secondary injury, represents a heterogeneously behavioral and cognitive deficit that remains incurable. Following SCI, various barriers containing the neuroinflammation, neural tissue defect (neurons, microglia, astrocytes, and oligodendrocytes), cavity formation, loss of neuronal circuitry, and function must be overcame. Notably, the pro-inflammatory and anti-inflammatory effects of cell–cell communication networks play critical roles in homeostatic, driving the pathophysiologic and consequent cognitive outcomes. In the spinal cord, astrocytes, oligodendrocytes, and microglia are involved in not only development but also pathology. Glial cells play dual roles (negative vs. positive effects) in these processes. After SCI, detrimental effects usually dominate and significantly retard functional recovery, and curbing these effects is critical for promoting neurological improvement. Indeed, residential innate immune cells (microglia and astrocytes) and infiltrating leukocytes (macrophages and neutrophils), activated by SCI, give rise to full-blown inflammatory cascades. These inflammatory cells release neurotoxins (proinflammatory cytokines and chemokines, free radicals, excitotoxic amino acids, nitric oxide (NO)), all of which partake in axonal and neuronal deficit.

Given the various multifaceted obstacles in SCI treatment, a combinatorial therapy of cell transplantation and biomaterial implantation may be addressed in detail here. For the sake of preserving damaged tissue integrity and providing physical support and trophic supply for axon regeneration, MSC transplantation has come to the front stage in therapy for SCI with the constant progress of stem cell engineering. MSC transplantation promotes scaffold integration and regenerative growth potential. Integrating into the implanted scaffold, MSCs influence implant integration by improving the healing process. Conversely, biomaterial scaffolds offer MSCs with a sheltered microenvironment from the surrounding pathological changes, in addition to bridging connection spinal cord stump and offering physical and directional support for axonal regeneration. Besides, Biomaterial scaffolds mimic the extracellular matrix to suppress immune responses.

Here, we review the advances in combinatorial biomaterial scaffolds and MSC transplantation approach that targets certain aspects of various intercellular communications in the pathologic process following SCI. Finally, the challenges of biomaterial-supported MSC transplantation and its future direction for neuronal regeneration will be presented.

## Introduction

Spinal cord injury (SCI) is a devastating disorder that affects approximately 18,000 new patients worldwide each year, causing both a high disability and mortality rate [1, 2]. SCI disrupts neuronal circuitry, causing permanent paraplegia or tetraplegia and other distinct implications to the patients (e.g., intractable pain, infections, and pressure sores), generating severe economic and social burdens on the people and their families. Traumatic injury to the spinal cord can be caused by compressions, lacerations, and contusions, which lead to a spectrum of neurological symptoms depending on the level and the severity of the injury such as motor/sensory dysfunction, autonomic deficits, neuropathic pain, autonomic dysreflexia, and bowel/bladder dysfunction [3]. The processes occurring within the SCI can be divided into acute (< 48 h), subacute (48 h to 14 days), intermediate (14 days to 3 months), and chronic phases (> 3 months).

Primary and secondary deaths are involved in the pathological process of nerve injury. The former is the direct death of nerve cells caused by immediate physical injury and is irreversible. The latter is caused by subsequent pathological changes, including necrosis, apoptosis, necrosis, autophagy, and pyrolysis. The pathological process caused by traumatic SCI involves vascular, neural, and immune systems, including the disruption of the blood supply, neuron death, a growth-inhibitory microenvironment, cyst formation, scar formation, and demyelination [4].

The traumatic injury damages the membranes of cells and causes primary injuries, which include cell death and blood vessel rupture [5]. Most neurotrophic drugs are widely used in experiments to alleviate secondary cell death, but because of the blood-brain and blood-spinal cord barrier, many drugs may not reach capable target cells. Thus, due to the particularity and complexity of the nervous system, no reliable neurotrophic drug has been identified.

In addition, there is a substantial cost burden associated with medical expenses each year for the treatment of nerve injury. Researchers are therefore trying to investigate effective neuroprotective measures, such as drug pretreatment [6], ischemic post/pre-conditioning or hypothermia [7], and exosomes [8]. However, the effects of these treatments are limited because the central nervous system is inaccessible to these treatments. Various nanomaterials, including liposomes [9, 10], polymeric micelles [11], carbon nanotubes [12], dendrimers [13], inorganic particles [14], and silica-based materials [15] have been used as targeted carriers. Recently, hydrogels hold promise for delivering treatments to nerve injury and targeting neurons to enhance axon regeneration and synaptogenesis. Drug delivery particles or tubes can extend the release of a drug but typically require a hydrogel to keep the biomaterial within the lesion cavity or to prolong release further. While hydrogels and particles/tubes do not effectively guide the regeneration of white matter tract axons, guidance conduits or fibers can effectively direct regeneration. Overall, an opportunity exists to develop drug delivery biomaterial approaches that provide aligned topography, appropriate mechanical characteristics, and specific drug release profiles to more successfully treat SCI.

Numerous studies have demonstrated that diverse biomaterials are potential delivery vehicle options for cells, bioactive molecules, and drugs in different models of SCI [16]. Biomaterial-based cell therapy transplant various cell types into the damaged area to address the issues of cell viability, cell retention at the lesion site, supportive physical matrix, filling of the lesion cavity, and mediating directed growth. Among the various types of stem cells, MSCs are commonly used for cell therapy owing to many advantages, such as their capacity for self-renewal and differentiation into multi-lineage without ethical issues. Moreover, MSCs advantageously have low immunogenicity and diverse sources. Biomaterials, such as hydrogels, provide a mimic of a three-dimensional (3D) in vivo microenvironment to maintain MSCs viability and to induce the MSCs fate to differentiate into multi-lineage.

The encapsulation of MSCs, by entrapping the viable cells in hydrogels, is considered to have the potential for the amelioration of micro-environment in the treatment of the nervous injury. Prior studies prove that MSCs’ encapsulation technologies maintain MSCs viability and function and provide optimal structural support for MSC infiltration and proliferation [17]. Then encapsulated MSCs can differentiate into neuron [18], intervertebral disc [19], bone [20], and articular cartilage [21]. Therefore, the encapsulation of MSCs into hydrogels for local cell secretion is a very effective and exciting strategy for the treatment of SCI.

### Different phases in neuroinflammation are critical for neuroprotection after SCI

Traumatic SCI can cause destructive events, such as neural cell death, tissue destruction, vascular rupture, and leakage, leads to an innate immune response characterized by persistent neuroinflammation (as a type of microglia activation) which exerts a crucial role in the pathological process of the secondary phase of SCI [22]. The expression of damage-associated molecular patterns (DAMPS), which are a diverse set of host molecules such as proteins and nonproteins, are responsible for initiating innate immune activation, including homing of inflammatory cells, after SCI [23]. However, neuroinflammatory processes appear as a double-edged sword by being either deleterious through the production of damage-associated molecular patterns (DAMPs) by neurons and glial cells which results in pro-inflammatory cytokines and chemokine production, blood-brain barrier (BBB) disruption, infiltration of leukocytes from the peripheral blood into the infarcted area, and further exacerbation of tissue damage or beneficial through promoting tissue repair process [24]. Immune cells also infiltrate an injury site through ruptured blood vessels to encounter the DAMP-positive microenvironment and exacerbate the inflammatory milieu [25]. Concerning SCI, DAMPs induce pro-inflammatory activation in microglia, astrocytes, and neurons. The pathological inflammation wave as part of secondary injury processes is critical to debris clearance, phagocytes are of dying neurons, and regulation of angiogenesis following SCI, particularly with unregulated inflammatory cytokine signaling [26, 27].

SCI is characterized both by the acute and focal contusion, as well as by an extensive secondary injury composed of ischemia, excitotoxicity, and uncontrolled neuroinflammation response [28]. The initial, acute phase of neuroinflammation is characterized by rapid infiltration of neutrophils, monocytes, and lymphocytes at the site of damaged tissue after a more extended period, accompanied by a continuous generation of cytotoxic molecules, including reactive oxygen species (ROS), reactive nitrogen species (RNS), proteinase 3, cathepsin G, and elastase5, which are likely to contribute significantly to secondary tissue degeneration [29]. Therefore, peripheral immune cells significantly and persistently contribute to the neuroinflammation microenvironment after SCI. After traumatic SCI, the role of circulating levels of the chemokines CCL2 and CXCL10 mediates macrophage migration and activation for repair in the subacute stage [30]. Macrophage chemoattractant protein (MCP-1) and other cytokines present in the damaged tissue promote recovery after SCI by altering macrophage polarity [31]. The cells, such as microglia, astrocytes, neurons, and oligodendrocytes, upregulate IL-1b and TNFa within hours [32]. After the initial inflammatory phase, induction of anti-inflammatory and reparative-phase is regulated by inflammatory protein and chemokine receptors in macrophage and regulatory T lymphocytes [33, 34]. Myelin debris, engulfed by microvascular endothelial cells, may promote macrophage recruitment and change phenotypic and functional of macrophage after neural injury [35].

In the secondary phase of neuroinflammation, lymphocytes drive incessant degeneration that occurs within several months. The infiltrating peripheral myeloid cells play an essential role in the secondary phase of SCI. A reduction in neutrophils present at the contusion site promotes recovery, while depletion of macrophages via clodronate liposomes reduces tissue damage. These myeloid cells may be driven toward a more inflammatory phenotype by the presence of cytokines such as TNF-a and IL-1b and free radicals. Besides, the spontaneous demyelination was caused by increased expression of TNF-α via activation of macrophages and microglia [36]. After SCI, there is no efficiently regulated induction of an anti-inflammatory and reparative phase, resulting in a chronic cytotoxic inflammatory state that contributes to secondary degeneration, thereby limiting repair and functional restoration [37, 38]. In the spinal cord, infiltrating monocytes and polarizing the differentiation of resident microglia toward an M2 or another reparative macrophage phenotype could promote restoration of SCI while restricting secondary inflammatory-mediated injury [26]. Examination of the spinal cord tissue showed the increased inflammatory cerebrospinal fluid biomarkers and persistent presence of inflammatory cells [39].

It seems that an elevated TNF-α level after SCI is responsible for initiating the neuroinflammatory response. In the initial period after SCI, TNF-α is produced by neurons and in a subacute period by microglia, astrocytes, lymphocytes, and macrophages derived from the peripheral blood. The increase of TNF-α in the blood and cerebrospinal fluid after the onset of SCI causes apoptosis of neurons and the increase of neurological deficits and induces the migration of leukocytes from the vascular bed to the vicinity of the lesion.

### Secretome of MSCs and suppression of neuroinflammation

A type of anti-inflammatory cell therapy is the transplantation of stem cells that activate downstream cellular pathways and promote the infiltration of endogenous NSC to the site of stroke injury. This involves the transplantation of stem cells, which have either been differentiated from iPSCs, ESCs, MSCs, or BMSCs to a neural progenitor state or are un-differentiated. Despite some differences in cell sources, MSCs still exhibit a remarkable autocrine and paracrine activity that can stimulate proliferation and differentiation of other cells and themselves, which is the dominating mechanism of MSCs’ participation in tissue regeneration and functional restoration following SCI. MSCs can secrete a variety of soluble molecules include tumor necrosis factor (TNF)-β1, interleukin(IL)-13, IL-18 binding protein, ciliary neurotrophic factor (CNTF), neurotrophin 3 factor (NT-3), IL-10-, IL-12p70, IL-17E, and IL-27 to exert anti-inflammatory potential [40]. Furthermore, the release of pro-inflammatory cytokines such as interferon, TNF, and IL-10 can also be inhibited by MSCs to a modulated cytokine production of the host.

Further, cells produce a wide variety of growth-promoting molecules, including brain-derived neurotrophic factor (BDNF), CNTF, glial-derived neurotrophic factor (GDNF), leukemia inhibitory factor (LIF), nerve growth factor (NGF), and neurotrophin 3 (NT-3), and ECM proteins like laminin, fibronectin, and collagen I/III and IV [41]. One of the essential methods by which MSCs secrete biological factors is via extracellular vesicles (EVs), which are divided into either microvesicles or exosomes. Extracellular vesicles are packed with thousands of proteins, 27 messenger RNA, and microRNA, many of which are enriched in EVs and have been reported to enhance neuronal growth in the SCI model [42, 43]. Given that much of MSCs paracrine actions are mediated via EVs, these subcellular packages are being developed as a cell-free biological therapeutic in their own right, which would obviate the theoretical teratogenic concerns of cell therapy. MSCs may improve neuronal health by donating their mitochondria [44]. This mechanism of mitochondrial transfer has been observed between astrocytes and neurons in a stroke model. Through this mechanism, MSCs conceivably could improve neuronal health by donating healthy mitochondria to neurons that harbordys functional mitochondria.

From a neurodegenerative perspective, it has become increasingly recognized that neuroinflammation plays a significant pathomechanistic role [45]. Neuroinflammation in this context is defined as the negative contribution of nonneuronal cells (e.g., immune cells, glial cells) to neurodegenerative disease. Many repair mechanisms rely on beneficial aspects of inflammation, yet certain aspects of inflammation cause damage to spared tissue. MSCs can also modify the immune response after injury by elevating levels of anti-inflammatory and reducing levels of proinflammatory cytokines when transplanted 3 or 7 days after SCI. The delivery of neural progenitor cells to the site of injury triggers recovery through reducing inflammation and reactive gliosis as well as promoting angiogenesis [46] without the heightened risk of tumorigenesis. Cell therapies are commonly administered through i.v. injection, requiring cells to cross the BBB. Much larger doses of cell transplantation are needed to treat the restrictions of selective permeability of the endothelium on cell infiltration. To circumvent this limitation, dual therapies including stem cells administered with the biomaterial, astrocyte-derived conditioned medium or drugs that transiently open the BBB have been considered [47]. Antagonizing the inductive effect of myelinoligodendrocyte glycoprotein (MOG), MSC-derived exosomes suppress neuroinflammation and show anti-neuroinflammatory and autoimmune suppressive characteristics after SCI. Also, MSC-derived exosomes decrease the frequency of inflammatory microglia in the damaged regions [48]. Therefore, MSC-derived exosomes may exert their beneficial roles in modulation of microglia through suppression of neuroinflammatory feedback. Notably, MSC migration can also be influenced by the release of cytokines, interleukins, and growth factors via an autocrine loop. Moreover, the MSC secretome can also exert immunomodulatory, anti-inflammatory, neurotrophic/neuroprotective, and angiogenetic effects on the host microenvironment [49].

However, maintaining the retention and stability of exosomes over time and resist of microenvironment variation in vivo after transplantation is a significant barrio to enhance the therapeutic efficacy in the clinical application of MSC-derived secreta [50, 51]. Tan et al. [52] demonstrated that therapeutic molecules such as neurotrophin (BDNF) could be effectively encapsulated and delivered to the target tissue of SCI in a sustained manner with retained biological activity via biomaterials, reducing undesirable biological side effects in non-target regions.

### Combination of biomaterial and MSCs improve the microenvironment

Biomaterial-supported MSC transplantation that includes the controlled and sustained delivery of therapeutic biomolecular and cell replacement offers a potential strategy to achieve tissue regeneration in the damaged region. Two main mechanisms are underlying this strategy for SCI repair, differentiation into neurons or glial cells for replacing the lost cells and secretion of neurotrophic factors for protection of neurons. Donor cells may replace lost glial cells and neurons, contribute to the re-establishment of new functional local circuits, and remyelination spared axons [53]. Besides, to encourage stem cell–scaffold interactions, many immobilized biological components are often used to decorate materials. In addition to the nature of the scaffold material, antibodies, protein modifications—including with epidermal growth factor (EGF), neurotrophin-3 (NT-3), and glial cell-derived neurotrophic factor (GDNF)—can be applied to the scaffold to regulate stem cell fate [54]. They can provide an avenue for continuous growth factor delivery, which can alter the environment, making it more conducive for regeneration. Biomaterials can provide sustained release and protect growth factors or other drugs from degradation, potentially avoiding wash away of transplanted cells [55].

The lack of neurotrophic factors and glial scarring, together with the neuroinflammation, is considered to be crucial targets for the reconstruction of functional connections [56]. Co-transplantation of biomaterial and MSCs, which are manipulated or genetically edited to express certain proteins, exerts neuroprotective, and anti-inflammatory effects to induce anti-inflammatory mechanisms [57]. Biomaterials themselves can have anti-inflammatory properties. For example, MSCs synthesize low levels of permissive growth factors and deliver a variety of neurotrophic factors to promote axonal regeneration [58]. Furthermore, MSCs show anti-inflammatory properties by interactions with host immune cells and producing immunoregulatory cytokines, including interleukins and TGF-β. In addition, MSC transplantation reduced reactive astrocyte proliferation and microglial cell activation. In addition to the low cell survival after transplantation, an increased neuroinflammatory response, characterized by microgliosis and astrogliosis, was observed after MSC transplantation in healthy CNS [59, 60]. Microglia can recognize the presence of autologous MSCs and become activated in an ideal way, demonstrated by the expression of inducible nitrogen oxide synthase.

In addition to providing a favorable microenvironment for establishment of functional neural connections, combination transplantation provides a potential therapeutic option for SCI that inhibits secondary glial scarring and ultimately promotes axonal regeneration. Inhibition of the glial scarring that impedes axonal regeneration is proposed to exert a beneficial effect on functional restoration. Biomaterial-supported MSC transplantation diminishes fibrosis during the early process of secondary SCI and further attenuates secondary glial scarring [61]. Subsequently, biomaterial-supported MSCs that were transplanted into the damaged region prevented the accumulation of CSPGs (the composition of a glial scar) and significantly promoted myelination of axon fibers and synapse formation. The MSCs can cooperate with biomaterials to support the growth of stem cells and endogenous neuronal cells by bridging the gap.

### MSC transplantation modulates the immune system

Extensive literature exists on the benefits of immune cells for CNS remyelination. In culture, Th2, rather than Th1, cells promoted OPC maturation [62]. The transfer of myelin-reactive Th17 cells was found to attenuate remyelination after demyelination, although the infiltration of myelin-specific T cells was shown to enhance oligodendrogenesis. Spontaneous remyelination of genetically deficient regulatory T cells was attenuated after SCI and was rescued by subsequent adoptive transfer of T regulatory cells, which deliver an oligodendrocyte trophic factor, CCN3 [63].

MSCs effectively modulate the immune system and can aid neurological restoration by mediating the immunomodulatory effects via direct cell–cell interactions [64, 65]. However, few studies dissect the direct interactions between transplanted cells and immune cells; instead, they measure global changes in cytokine release or the abundance of immune cells after SCI. The immunomodulation is realized thanks to the expression of the major histocompatibility complex-I on the MSC surface, in this way preventing T cell recognition and induction of a host immune response. Unlike most other allogeneic cell therapies in clinical development, allogeneic MSC therapies may be used without concomitant immunosuppression because of their paucity of dominant histocompatibility complex class II proteins and decreased propensity to trigger an immune response [66, 67]. Moreover, MSCs can inhibit the proliferation, activation, and differentiation of T cells. Besides, MSC transplantation can provide benefits through immunomodulation via the production of different cytokines, growth factors, and immunomodulatory factors (for example, nitric oxide, indoleamine 2,3-dioxygenase, TGFβ) that can inhibit T cell proliferation, induce tolerogenic dendritic cells or prevent/polarize T cell activation [68]. Transplanted cells might alter inflammation indirectly by dampening tissue damage. In addition, MSCs modulate neurodegenerative disorders via the paracrine conversion of CCL2 from agonist to antagonist of CD4 Th17 cell function [69]. For example, MSC transplantation robustly decreased CD4 T cell infiltration of the spinal cord along with reduced plasma levels of IL-17 and TNF level. Both allogeneic and autologous MSC therapies are in development.

### Communication between macrophages and MSCs

Extensive research has shown that the behavior of MSCs has immunoregulatory potential following activation by macrophages [70, 71]. The directionality of the communications between macrophages and MSCs reveals a strictly controlled reciprocal relationship for modulation of tissue repair and regeneration. Previous research has established that macrophages promote healing in various tissue types via bidirectional crosstalk with MSCs [72, 73].

#### Macrophages influence MSCs

Macrophage-derived cytokines and inflammatory environment influence the MSC secretomes, migratory, and immunomodulatory capacity [71, 74]. Peng et al. demonstrated that HIV-1-infected and immune-activated MDM could affect neurogenesis through induction of NPC proliferation, inhibition of neurogenesis, and activation of gliogenesis [75]. Redondo and colleagues found interleukin-1 primes MSCs toward an anti-inflammatory and pro-trophic phenotype in vitro [76]. Additionally, Hayakawa et al. showed that CD200 restrains macrophage attack on oligodendrocyte precursors via Toll-like receptor four downregulation in the nervous system [77].

More importantly, this work introduces the potential of ILP/ISP as a viable strategy for modulating the immune response following SCI and other neuroinflammatory conditions of the central nervous system. Considering the sequence of macrophage phenotypes during SCI, it is clear that the potential of inflammatory cytokines in the microenvironment was introduced as a feasible strategy for modulating the immune response and neuroinflammatory conditions [78]. Further research into anti-inflammatory and immunomodulatory mechanisms of MSC transplantation could promote the development of therapies that harness the reparative and modulatory potential of macrophages and MSCs to construct the reparative microenvironment for the SCI.

#### MSCs modulate macrophage polarization

On the other side, the accumulated evidence in the literature so far reveals that MSCs support a shift among macrophages from the M1- to M2-like phenotypes. It was found that in vitro MSC secretomes stimulate the migration of RAW264.7 macrophages and recruit immune cells in the spinal cord of mice 1 to 7 days after SCI [79]. Various immunosuppressive cytokines secreted by MSCs switch macrophages to anti-inflammatory phenotypes by inducing modifications in the metabolism of macrophages [80, 81].

MSC-conditioned medium favors tissue repair by inducing the M1-to-M2 switch, and enhancing M2 macrophage features, reducing the secretion of the inflammatory cytokines, IL1β, IL6, and TNFα [82]. Macrophages in culture with MSC spheroids, which secrete enhanced levels of PGE2, or with conditioned medium thereof, polarize from M1-like to M2-like macrophages [83]. Systemic administration of MSC-derived exosome possesses practical anti-inflammatory effects by endorsing anti-inflammatory macrophages [84].

Transplantation of MSCs into the contused spinal cord in adult rats was found to result in a decrease in TNF-α, IL-6, and IL-1β and an increase in IL-4 and IL-13 accompanied by tissue sparing, axon preservation, decreased scar formation, and improved functional outcomes [85]. Treatment of the adult mice contused spinal cord with conditioned medium of embryonic MSCs resulted in the restoration of the macrophage phenotype after the inflammatory phase and improved locomotor recovery, indicating that soluble factors are essential in MSC immunomodulation. The scaffold could efficaciously deliver CCL2 secreted by human MSCs, which acts a part in not only recruiting macrophages but also driving their transformation to an M2 neuroprotective phenotype, to preserve motor neurons and myelin in the lesion [86].

### Communication between microglia and MSCs

In general, brain injury results in microglial activation (either classical or alternative), which can be beneficial or detrimental for different types of CNS diseases [52]. Directed modification of the microglial activation status could positively influence the disease course, thereby offering great potential as a therapeutic strategy. Moreover, this approach also holds promise for altering the immunological responses observed after stem cell transplantations, intending to decrease neurodestructive immune responses at the site of injection [87]. In order to use the adequate approach (stimulation or inhibition of neuroinflammation and neuroprotection), it is imperative to start by defining the activation status of microglia and their function in CNS disease or following stem cell application. The exact specification of the type of microglia activation may prove challenging, as it is highly dependent on the type of stimulated PRR, the location of microglia in the CNS, and the time point during the disease course. Applying this concept to an example such as stroke, cytotoxic microglia is observed in the striatum, whereas subventricular zone microglia promote neurogenesis [53]. Furthermore, mouse models for amyotrophic lateral sclerosis exhibit neuroprotective microglia at disease onset, which is gradually transformed into cells with a more neurotoxic phenotype at the end of the disease [88]. Therefore, therapeutic intervention at the right place and time is vital to achieving success. Strategies can either focus on the inhibition of microglia activation by maintaining tissue homeostasis or the modification of the activation status (classical or alternative activation or acquired deactivation) of microglia in order to attain neuroprotection. Based on the complex microglial activation signaling, modulation of neuroinflammation can be exerted at different levels of the signaling pathway: PPARs, TLRs, NF-κB signaling, JAK/STAT signaling, ATP signaling via purinoreceptors [89].

The microglia plays a role in regenerative functions for supporting the regeneration of myelin after SCI, a process that is critical for axonal health. How remyelination is controlled and how microglia are involved are not entirely understood. Both the M1- and M2-like macrophage/microglia phenotypes have roles in remyelination. In a postdemyelination state in animals, pro-inflammatory (M1) macrophages/microglia predominate at early stages, and the depletion of these cells impairs OPC proliferation; at later stages of postdemyelination, regulatory (M2) macrophages/microglia predominate, and the depletion of these cells inhibits OPC differentiation [90]. The types of molecules affected by the predominant subset in a particular injury microenvironment is likely an important determinant of whether overall harm or beneficial results.

Mediators of inflammatory response such as cytokines and chemokines are also observed in the lesion area. These cytokines and chemokines are secreted by activated cells present in the spinal cord tissue, such as neurons and neuroglial cells, and peripheral blood cells after an occurrence of hypoxia-ischemia. Interleukin-1 (IL-1) is the primary inflammatory cytokine mainly produced by microglia, astrocytes, and neurons following SCI [91]. In addition, it affects endothelial cells mainly by increasing the expression of intercellular adhesion molecule 1 (ICAM-1) and vascular cell adhesion molecules 1 (VCAM-1), which initiates neutrophil penetration into the damaged tissue [92]. In the spinal cord tissue, IL-6, produced by microglia, macrophages, astrocytes, and neurons, was regarded as a pro-inflammatory cytokine and was conducive to the SCI by activating lymphocyte and acute-phase proteins [93]. Neuroprotective IL-6 enhanced to strengthen the activity of IL-1, and IL-1 conversely inhibited its proinflammatory effect through its receptor antagonist (IL-1Ra) [94].

Intraspinal transplantation of MSCs 1 week after SCI is associated with a polarization of macrophages toward an M2 phenotype and away from an M1 phenotype, which is correlated with increased spared white matter55. Despite a lack of cell entry into the spinal cord, intravenous delivery of MSCs 1 day after contusive SCI is associated with increased forelimb–hindlimb coordination and improved micturition, which correlates with increased spared tissue and an elevation in M2 markers57. There is also decreased proinflammatory cytokine production in the spleen and blood, indicating a systemic change in the inflammatory state. Importantly, there is no need for neural integration of MSCs: conditioned medium from MSCs can improve motor function after SCI, suggesting that cell transplantation may not be required to achieve functional benefits. Prior study has demonstrated that MSCs make less microglial infiltration and decreased levels of IL-6, TNF-α, and p-STAT 3 in dogs with acute SCI [95]. Hideaki et al. suggested that MSC transplantation modified the inflammatory microenvironment by shifting the macrophage phenotype from M1 to M2 and that this may significant increases in IL-4 and IL-13 levels, and reductions in TNF-α and IL-6 levels [96]. The cytokines produced by MSCs affect neuroinflammation, both on the level of inhibiting microglial activation as well as modulation of microglia activation. Under steady-state conditions, microglia display a ramified morphology and exert surveillance of the CNS in order to accurately respond to danger signals [97]. Different mechanisms involving microglia-neuron signaling can maintain these steady-state conditions, while the disruption of this cross-talk results in microglial activation [98]. Activated microglia, astrocytes, and T cells can interact and increase neuronal death due to proinflammatory and reactive oxygen species production [99, 22, 23]. Interestingly, MSCs may be either anti-inflammatory or proinflammatory, depending on the milieu within which they exist. When entering an inflammatory milieu (interferon-g, tumor necrosis factor-a), MSCs become anti-inflammatory, wherein they secrete transforming growth factor b1, indoleamine 2,3-dioxygenase, and prostaglandin E2 and can convert macrophage/microglia from the pro-inflammatory M1 to the anti-inflammatory M2 phenotype [100].

### Communication between astrocytes and MSCs

After SCI, astrocytes undergo significant morphological, molecular, and functional changes represented by hypertrophy, increased proliferation, and upregulation of intermediate filaments glial fibrillary acidic protein (GFAP), vimentin, and nestin—hallmarks of reactive astrogliosis. Furthermore, reactive astrogliosis is known for the production of proteoglycans, various cytokines, and chemokines. Notably, astrocytes exist in two distinct reactive states, such as A1 neuroinflammatory reactive astrocytes, of which complement component C3 is specifically upregulated, and ischemia-induced A2 neuroprotective reactive astrocytes [101]. A1-reactive astrocytes, induced by microglial activation after SCI, cause death of axotomized neurons [102]. The astrocyte activation and the glial scarring at the lesion site remain a barrier for axonal regeneration and the remyelination of uninjured axons following SCI. For example, Liu et al. were the first to demonstrate that intravenous injection of MSC-derived exosome after SCI leads to a decreased number of C3 + /GFAP + astrocytes in the damaged region in acute period. They ascribe these results to the secretion of TSG-6 by MSCs and consequently decreased NF-κB signaling, which induces А1 neuroinflammatory reactive astrocytes [103, 104]. Studies designed to estimate the efficacy of MSCs is intended for neuroregeneration in SCI concentrate on the state of reactive astrocytes and extracellular matrix in the glial scar. Typically, the role of the glial barrier and reactive astrocytes is considered a negative one despite a known decisive role in maintaining the tissue structural integrity and the process of neuroregeneration during an early post-SCI period [105, 106]. Studies with biomaterial-supported MSC transplantation after SCI provide evidence to reduced glial scar and astrocyte activation both in acute [107], subacute [108], and chronic periods following SCI [109]. The decreased expression level of GFAP and less that of proteoglycans are estimated in the damaged region, which means that the glial barrier is reduced and axonal growth results [110, 111]. However, in addition to the perplexing and multicomposition structure such as reactive microglia/macrophages and astrocytes, microglia precursors, fibroblasts, and perivascular cells made up the glial barrier, penetrating into a scar [112].

Transplanted MSCs typically differentiate into astrocytes, and only a small portion of MSCs give rise to neurons or oligodendrocytes [113, 114], with some neurological restoration being observed. Pluripotent cells are differentiated into specific neuronal lineages (such as motor neurons) or progenitor cells are combined with neurotrophic proteins to promote directional growth to replace and integrate neurons. Besalti et al. demonstrated that an early intravenous administration of allogenic MSCs would upregulate the expression of GFAP by 7 days after injury [115]. MSC transplantation contributes to the formation of a neuroprotective microenvironment by GFAP-expressing astrocytes for neurogenesis [47]. It has been shown that reactive and hypertrophic astrocytes start expressing COX-2 within the area of ischemia in response to pro-inflammatory stimuli [116]. The possibilities of preventing astrocytosis are attributed to the ability of transplanted MSCs to decrease cyclooxygenase-2 (COX-2) [96, 117] and IL-6 cytokine levels [118]. In animal models of SCI, culture conditions of transplanted MSCs, such as neurotrophin-3 and brain-derived neurotrophic factor [BDNF], in combination with glial progenitor cells have been demonstrated to promote functional restoration. For example, astrocytes derived from glial progenitor cell-specific culture conditions, such as bone morphogenetic protein 4, exert beneficial effects in SCI. The restricted range of cell types and the limited availability of multipotent neural progenitor cells may limit their utility. Whether transplanted MSCs can migrate to a distant location from the lesion site and be readily differentiated into neurons and astrocytes remains controversial. Therefore, we do not anticipate that transplanted MSCs would mediate any beneficial effect by incorporating into the lesion site of spinal cord to replace dying neurons, which may be effective in neural stem cell therapy.

While the biomaterial scaffolds and MSC transplantation have each reported beneficial effects as monotherapies, many studies demonstrated the synergistic enhancements elicited by combinational therapy. When injected into the SCI, this combined strategy inhibited neuronal cell apoptosis, neuroinflammation, and astrogliosis. The combinational of biomaterial injection and MSC transplantation improved MSC engraftment, reduced astrogliosis, and glial-secreted chondroitin sulfate proteoglycan (CSPG) deposition, and increased synaptic connectivity compared to monotherapies [119]. There is also a considerable possibility for an implanted or injected material to reduce astrocyte reactivity and inhibitory CSPG deposition, either through intrinsic material properties or the release of therapeutic agents. However, without the use of a carrier device (e.g., hydrogel), the lifespan and localization of MSCs are limited (transfusions of MSCs tend to localize to the lung tissue).

### Oligodendrocytes and Schwann cells: development and role following MSC transplantation

In the context of SCI, functional capacity is lost due to the death of neurons and oligodendrocytes. Regeneration of adult CNS neurons is not a one-step process rather, and it requires that the neuron survives (inhibits apoptosis), extends its processes toward its original neuronal target (site-directed migration), remyelination, and forms functional synapses. MSCs can differentiate into neurons, astrocytes, and oligodendrocytes upon exposure to physical and chemical cues. Although CNS fibers regenerated into the grafts, they failed to re-enter the host spinal cord [120]. In addition, the corticospinal tract (CST), which is responsible for the majority of voluntary movement, failed to regenerate and enter into Schwann cell or peripheral nerve grafts. Attempts to overcome the immunogenicity while maintaining its core function include site-directed mutagenesis. Cell-material hybrids elicit an adaptive immune reaction that further influences the host response to the material [121].

Combinatorial strategies positively influence the poor microenvironment for cell survival, neuronal differentiation, and maturation following SCI., which is an area of intense research.

Myelin sheaths are distinctive membranes with high lipid content and few proteins that surround the axons, protecting them by releasing trophic factors and increasing signal transduction velocity. Myelin is an essential neural circuit structure whose regeneration remains a challenging issue in SCI [122]. Moreover, secondary demyelination is caused by myelin degradation due to neuronal or axonal loss. Within 24 h of spinal cord trauma, neuron and myelin debris and loss of myelin proteins have been observed in areas of white matter damage, and myelin debris accumulates within the first week of injury and persists in the chronic SCI. The sustained presence of myelin debris inhibits axon regeneration, oligodendrocyte differentiation, and remyelination. Following a demyelinating incident, remyelination does not occur from existing myelinating oligodendrocytes but from a reservoir of previously quiescent and mature NG2 glia, which differentiate into new myelinating oligodendrocytes. Activated microglia and astrocytes are responsible for prompting recruitment, proliferation, and transformation of inert precursor cells into differentiating NG2 glia in neuroinflammatory conditions. Demyelination and axonal injury, a part of a process of secondary degeneration, remyelination employ mature oligodendrocytes which originate from OPC to create new myelin sheaths around the axons [123]. Axon myelination facilitates neuronal performance by increasing the speed of nervous impulse, and therefore, damage to myelin and oligodendrocytes can cause severe dysfunction [124]. Like macrophages/microglia, oligodendrocytes are also implicated in remyelination. An examination of these samples documented that the density of O4-positive oligodendrocyte precursor cells was associated with the density of macrophages/microglia. Oligodendrocytes succumb to apoptotic or necrotic death via several post-SCI processes: ischemia and oxidative damage, with oligodendrocytes being particularly sensitive; glutamate- or ATP-elicited excitotoxicity, which can elicit in oligodendrocyte toxic accumulation of exorbitant calcium; pathologic lipid signaling, which can activate cell death pathways; and an extreme inflammatory environment, which includes cytokines that can kill oligodendrocytes directly or indirectly. Oligodendrocytes in the spinal cord are responsible for generating myelin. Oligodendrocyte precursor cells (OPCs) proliferate, differentiate, and migrate to damaged sites in order to remyelinate in response to demyelination events, but this procedure usually is faulty [125]. OLs provide axonal support via insulating myelin, but they also serve a broader role. It is possible that OLs provide metabolic support to axons all along their length. The diffusion barrier to metabolites posed by the myelin, as well as an absence of glycogen in OLs rules out an isolated glycolysis support mechanism, and in addition, it was found that certain parts of the myelin sheath that wrap the axon were formed by NSC-derived myelinating cells. Stimulated by various factors, oligodendrocytes upregulate proinflammatory cytokines, cytokines, IFN-γ, and several chemokines. These immune receptors and cytokines could modulate activation of adjacent glial cells [126]. In a rat model of noncompressive lumber disk herniation-induced spinal cord damage, IL-33/ST2 expression in the oligodendrocytes modulates MAPK and NF-κB activation and inflammatory mediator expression [127].

Within 24 h after a contusion injury, necrosis/hemorrhage induced-demyelination occurs in primary white matter due to direct insult is observed. Similarly, humans with chronic SCI present with variable amounts of demyelination in the lesion penumbra [205]. Therefore, demyelination of spared axons in the lesion border could restrict axon conduction and contribute to post-SCI functional deficits. It is also possible that benefits following myelinating cell transplantation are conferred by mechanisms other than remyelination. Transplantations with enhanced oligodendrocyte differentiation over naïve NPCs have been associated with improved myelination and locomotor function, indicating forming oligodendrocyte lineage cells may be particularly advantageous. Oligodendrocytes also provide trophic signals to nearby neurons and synthesize defined growth factors. Whether oligodendrocytes enhance spared white matter via direct trophic support of axons remains uncertain. Transplanted cells may also spur endogenous cell remyelination [128], which may be causal in locomotor improvements.

Combinations that target the host rather than the cell graft ideally target an aspect of the injury that is not already influenced by the transplant to maximize the effect of the combinatorial strategy. Hence, a multitude of regenerative (cell growth and survival) as well as non-regenerative (physical and biochemical) events need to function in tandem to restore functionality of the damaged neuron. While biomaterials have had widespread use in an attempt to promote neural regeneration within the injured central and peripheral nervous system, oligodendrocyte regeneration using similar material-based approaches are not as common or understood. The chemical composition of the substrate can have distinct modulating effects on NG2 glial maturation and differentiation. The MSC transplantation into the damaged area with the aim of producing new oligodendrocytes or Schwann cells has been frequently pursued to improve remyelination. Human ESC-derived oligodendrocyte progenitor transplants were able to restore locomotor function after SCI, which was potentially related to remyelination of spared axons. MSCs are transplanted into the lesion and differentiate into neurons and glial cells [22]. For example, chitosan-based biomaterials have been used in the past to promote oligodendrocyte survival and attachment [234], as well as differentiation into oligodendrocytes [234–237]. Materials that influence oligodendrogenesis from oligodendrocyte precursors include fibrin, HA-methylcellulose, poly (lactic-co-glycolic) acid (PLGA), and methacrylimde, while materials that promote myelination from mature oligodendrocytes include fibrin, PLGA, chitosan, and HA-gelatin [238]. Replacement of lost or damaged neurons or oligodendrocytes, which can facilitate myelination of spared axons, is an essential goal of stem cell transplantation. In addition, MSC transplantation facilitates myelination by producing soluble factors [129]. For example, reducing the expression of anti-oligomeric dendrogenic determinant Id2 and increasing the expression of pro-oligomeric dendrogenic factor Olig2 in neural progenitor cells, MSCs induce oligodendrogenesis by producing microRNAs and delivering them via exosome [130, 131]. MSC-derived soluble factors have been previously shown to prime neural stem/progenitor cells (NSCs) toward oligodendroglial fate by reducing the anti-oligodendrogenesis determinant Id2 and increasing the pro-oligodendrogenic factor Olig2 expression [130]. It was shown in vitro that MSCs could not only direct proliferating NPCs toward an oligodendrocyte fate but also induce oligodendrocyte differentiation. An intravenous injection of MSCs in the early post-injury period elevated the expression of GalC, regulating oligodendrocytes differentiate into mature oligodendrocytes [131]. However, the expression of an oligodendrocyte transcription factor (Olig2), which regulates oligodendrocyte differentiation does not seem to change, since the mRNA analysis of the Olig2 gene shows no significant differences with control groups.

The influx and proliferation of Schwann cells can also be attributed to MSC secretion of BDNF, vascular endothelial growth factor (VEGF), and other unknown inducing factors. BDNF was shown to promote a significant expansion in the number of Schwann cells at 3 weeks after SCI, with VEGF stimulating their proliferation. In co-culture, the MSCs lead not only to improved survival and proliferation of Schwann cells but promote increased expression of BDNF, nerve growth factor (NGF), and its high- and low-affinity receptors (TrkA and LNGFR) in these cells [132, 133]. MSC transplantation could elicit the influx of Schwann cells into the astrocyte-devoid lesion site and improve their survival rate. Numerous trophic factors, such as NGF, BDNF, GDNF, and CNTF, are secreted by transplanted MSCs [134], while can also remyelinate injured axons [135], facilitate the SCs into the SCI segments [136], or guide regenerating axons [137]. However, it seems that the transplanted SCs can promote the growth of axons to the grafted mass, when axons have to re-enter the caudal or rostral spinal cord tissue outside the transplant, due to the inhibition of peripheral glial scar caused by a strong astrocyte response and increased synthesis of CSPG. Transplantation of Schwann cells, OPCs, or NSPCs capable of producing oligodendrocytes can be used to enhance myelin regeneration after SCI. Transplanted OPCs or NSPCs are likely to compete with endogenous remyelinating cells for denuded axons and thus remyelination as a therapeutic approach after SCI is contentious. The differentiation of transplanted cells into oligodendrocytes does not always result in improved motor recovery, mainly if the level of remyelination is not sufficient to promote an overall increase in the number of myelinated fibers112. Transplanting Schwann cells in the acute or subacute setting after rodent thoracic SCI increases the number of axons with peripheral myelin and is often correlated with modest improvements in forelimb–hindlimb coordination during open-field testing. Transplanted Schwann cells can myelinate axons and enhance endogenous Schwann cell myelination. At the same time, Schwann cells were shown to migrate from peripheral nervous structures resulting from a disruption of the barrier integrity, to participate not only in remyelination of central axons in the area of injury but also in the restoration of conduction [138–141]. Therefore, it is not known to what extent spared axons are remyelinated by transplanted Schwann cells, nor is the contribution of this myelin to functional improvements proven. Transplantation of Schwann cells incapable of producing myelin, such as cells derived from trembler (Pmp22Tr) mutant mice, may be useful in establishing a causal relationship between myelin regeneration and functional improvements. Several MSC transplantations demonstrate an increase of myelin retention and the number of myelinated axons in the lesion site during a chronic post-injury period [57]. Thus, Schwann cell coculture improves the therapeutic effect of bone marrow stromal cells on recovery in spinal cord-injured mice. SC grafts may be considered for the focal reconstitution of a gap created in the injured spinal cord, but reproducible studies testing the combination of therapies involving SC transplants are still needed to assess their potential for a comprehensive SCI repair outcome.

### MSC transplantation stimulates angiogenesis, neurogenesis, and synapse formation

The mechanisms that underlie the beneficial effects of MSC transplantation include differentiation into neuronal and endothelial lineage as well as the induction of neurogenesis, angiogenesis, and synapse formation in rodents [142]. There is widespread blood vessel loss after SCI, which induces local hypoxia within and adjacent to the injury site. The proliferation of endothelial cells, or angiogenesis, occurs as early as third and stops by 10 days after SCI, but only after 14 days does vessel perfusion recover to baseline levels. MSCs can also induce angiogenesis, an essential process by which new vasculature sprouts from pre-existing blood vessels; to this aim, MSCs secrete the tissue inhibitor of metalloproteinase-1, vascular endothelial growth factor, platelet-derived growth factor (PDGF), IL-6, and IL-8. The production of these factors is particularly important for supporting wound healing processes.

Transplant-derived secreted molecules might promote blood vessel protection and regeneration. MSC transplantation restores function by repopulating the necrotic cavity present within the ischemic region [143]. Both histology and MRI revealed that the beneficial effects of MSC therapy result from improved revascularization in injured ischemic tissues [144]. Fetal tissue grafts re-establish oxygen levels within the graft and adjacent to the injury site39, which is correlated with elevated blood vessel density in and around the transplant site.

Angiogenesis that is induced by MSC transplantation promotes endogenous neurogenesis, which could enhance functional rehabilitation after ischemia-reperfusion injury in rats with SCI [145]. Newly studies demonstrated that MSC-derived exosomes promoted neurogenesis in animal models of SCI. MSCs were manipulating transcriptional regulators, supplementing various types of growth factors, and acting intervention on pharmacological pathways targeting diverse aspects of the endogenous neurogenic responses that influence adult neurogenesis following SCI. In addition, several latest studies indicated that MSCs effectively promoted neurogenesis in experimental models of SCI. However, the predominant mechanisms by which MSCs participate in neurogenesis after SCI are not their cell replacement effects but likely their secretion-based paracrine effects. To exert neuroprotection, MSCs secrete several neurotrophic factors, such as brain-derived growth factor (BDNF), glial-derived growth factor (GDNF), nerve growth factor (NGF), NT-1, NT-3, CNTF, and primary fibroblast growth factor (bFGF); through these factors, MSCs can, on the one side, prevent nerve degeneration and apoptosis, and, on the other, support neurogenesis, axonal growth, remyelination, and cell metabolism. However, the concrete cellular and molecular mechanism of this neurogenic process are still unclear. Indeed, prior studies have shown that MSC transplantation protects against ischemia injury by upregulating IL10 expression [146], suggesting the activation of endogenous neurotrophins.

### Conclusions and future perspectives

In the current review, we have outlined controlled stem cell therapies using biomaterial focusing on MSC transplantation. After a brief description of the multiple cellular roles, such as macrophages, microglia, astrocytes, oligodendrocytes, and their communication with MSCs after SCI, the design and development of new biodegradable materials capable of entrapping stem cells and therapeutic drugs could enhance this combinatorial approach, providing the highest chance of tissue renovation and maximal functional restoration in the clinic. We speculated that the therapeutic combination of biomaterials and MSC transplantation would positively improve their performance in SCI.

Recently, significant progress has been made to elucidate the pathophysiology of SCI and to uncover the mechanisms that make the injured spinal cord refractory to regeneration. Despite an incredible body of knowledge, few studies on the strategy that combines many different approaches, such as the combination of biomaterials and MSC transplantation in SCI, have been conducted, which offers opportunities for tissue repair and regenerative medicine. As more biomaterials are developed and combined with different treatment strategies, researchers should investigate how biomaterial-based treatments alter the progression of subsequent events, which include macrophage activation and inflammation, reduce oxidative stress, restore the BSCB, reduce glial scar inhibition, and guide neurite outgrowth. Various materials address multiple areas of secondary damage, which can reduce inflammation in neural electrodes and increase neurite outgrowth. By modulating inflammation, repopulating lost neural cells through transplantation, improving the local environment by implanting biomaterial scaffolds with growth factors, and implementing strategies to break down the inhibitory barriers, impressive recovery has been demonstrated in animal models of SCI. The efficacy of these combined strategies has been explored in vitro and in vivo models showing reductions in neuroinflammatory responses and promoted functional restoration, which has shown greater therapeutic effectiveness than those involving single treatments. This highlights our incomplete understanding of CNS physiology following injury, which poses a massive barrier to developing therapeutic strategies for complete functional regeneration.

Promotion of axon regeneration and cell replacement after SCI utilizing biomaterial scaffolds has been addressed through a variety of strategies. Embedding and subsequent secretion-based paracrine effects of MSC cell release, the use of neurotrophic factors, and small drug molecules are a unique tactic to overcome graft rejection. Beneficial properties such as biocompatibility, long-term stability in vivo, or their suitability to be chemically modified with appropriate ligands, make these materials promising scaffolds for embedding drugs, nerve cells, growth factors, etc. The combination provides a transplanted microenvironment, mimicking the MSCs niche in vivo, to influence the intercellular communication through biophysical signals from biomaterials and further facilitate tissue renovation. These pre-clinical studies build the scientific foundation to progress accelerate the translation of MSC transplantation therapies toward clinical applications. In particular, the use of biomaterials as anti-inflammatory agents—or as vehicles for controlled release of anti-inflammatory agents—in the form of NPs or hydrogels present as attractive candidates for promoting the efficacy of SCI therapies. The development and administration of biomaterials with appropriate physical properties in combination with MSC transplantation to treat post-SCI inflammation are crucial. Astrocyte and microglial activation result in distinct functions by being activated in different ways after autologous transplantation of MSCs into the SCI.

## Data Availability

Not applicable.

## Abbreviations

CNS: Central nervous system
SCI: Spinal cord injury
MSCs: Mesenchymal stem cells
SDF-1α: Stromal cell-derived factor-1α
NO: Nitric oxide
DAMPS: Damage-associated molecular patterns
BBB: Blood-brain barrier
ROS: Reactive oxygen species
RNS: Reactive nitrogen species
MCP-1: Macrophage chemoattractant protein
CNTF: Ciliary neurotrophic factor
TNF: Tumor necrosis factor
BDNF: Brain-derived neurotrophic factor
GDNF: Glial-derived neurotrophic factor
LIF: Leukemia inhibitory factor
NGF: Nerve growth factor
EVs: Extracellular vesicles
MOG: Myelin oligodendrocyte glycoprotein
EGF: Epidermal growth factor
ICAM-1: Intercellular adhesion molecule 1
VCAM-1: Vascular cell adhesion molecules 1
GFAP: Glial fibrillary acidic protein
COX-2: Cyclooxygenase-2
CST: Corticospinal tract
